# Histones bundle F-actin filaments and affect actin structure

**DOI:** 10.1371/journal.pone.0183760

**Published:** 2017-08-28

**Authors:** Edna Blotnick, Asaf Sol, Andras Muhlrad

**Affiliations:** 1 Department of Medical Neurobiology, Institute for Medical Research-Israel–Canada, Hebrew University of Jerusalem, Jerusalem, Israel; 2 Institute of Dental Sciences, Hebrew University-Hadassah School of Dental Medicine, Jerusalem, Israel; Semmelweis Egyetem, HUNGARY

## Abstract

Histones are small polycationic proteins complexed with DNA located in the cell nucleus. Upon apoptosis they are secreted from the cells and react with extracellular polyanionic compounds. Actin which is a polyanionic protein, is also secreted from necrotic cells and interacts with histones. We showed that both histone mixture (histone type III) and the recombinant H2A histone bundles F-actin, increases the viscosity of the F-actin containing solution and polymerizes G-actin. The histone-actin bundles are relatively insensitive to increase of ionic strength, unlike other polycation, histatin, lysozyme, spermine and LL-37 induced F-actin bundles. The histone-actin bundles dissociate completely only in the presence of 300–400 mM NaCl. DNA, which competes with F-actin for histones, disassembles histone induced actin bundles. DNase1, which depolymerizes F- to G-actin, actively unbundles the H2A histone induced but slightly affects the histone mixture induced actin bundles. Cofilin decreases the amount of F-actin sedimented by low speed centrifugation, increases light scattering and viscosity of F-actin-histone mixture containing solutions and forms star like superstructures by copolymerizing G-actin with H2A histone. The results indicate that histones are tightly attached to F-actin by strong electrostatic and hydrophobic forces. Since both histones and F-actin are present in the sputum of patients with cystic fibrosis, therefore, the formation of the stable histone-actin bundles can contribute to the pathology of this disease by increasing the viscosity of the sputum. The actin-histone interaction in the nucleus might affect gene expression.

## Introduction

Actin is the most important structural protein in the cell with multiple functions [1]. There are two forms of actin: the globular G-actin, which is monomer and the polymer F-actin, both forms are negatively charged. In the cell actin exists mainly in F-form. Monovalent, divalent and polyvalent cations including cationic proteins and peptides polymerize G-actin to F-actin. Positively charged polycationic proteins and peptides bundle F-actin via non-specific electrostatic interactions [2] by countering the repulsion between negatively charged actin filaments [3]. LL-37 [4], lysozyme [5] MARCKS [6], ENA/VASP [7], fesselin [8], and calponin [9] are such actin bundling proteins. We studied recently the bundling process of F-actin by lysozyme, spermine, polylysine [2], and LL37 [10], [11], [12]. There are also other actin binding proteins [13], [14], which bundle actin filaments by specific hydrophobic interaction via attachment to two actin protomers located on to two separate filaments. These proteins induce formation of tightly packed bundles containing parallel arranged filaments [14]. Bundling of actin filaments may have a role in the pathogenesis of cystic fibrosis [15]. In addition F-actin forms rod like superstructures with oxidized cofilin [16], which may have central role in the pathogenesis of Alzheimer disease [17]. Actin also present in the nucleus. The nuclear actin also exist in monomer and polymer forms [18], which have well defined functions, including gene expression regulation through transcription factors, chromatin remodeling [19], histone deacetylase activity regulation [20] and nuclear integrity maintenance [21].

Histones are highly charged polycationic small proteins located in the in eukaryotic cell nuclei. They package and order DNA into structural units called nucleosomes. Histones are the main protein components of chromatin, spool around DNA, and play a role in gene regulation. There are five major families of histones: H1/H5, H2A, H2B, H3 and H4. H2A, H2B, H3 and H4 are the core, while H1 and H5 are linker histones. All core histones contain the histone fold domain containing three alpha helices linked by two loops [22]. They are secreted from neutrophils and form neutrophil extracellular traps (NET) with other proteins and DNA and increase plasma clot formation [23]. Histones are also secreted from cells following apoptosis. Extracellular histones have various functions. They have antimicrobial activity [24], [25], [26] [27] and a role in the host defense system [28]. However, they act also as inflammatory agents synergistically with other secreted compounds [29]. Histones are major mediators of death in sepsis [30], [31] and they have a significant role in tissue injury and inflammation [32]. Histones were shown to interact with actin. H1 histone was found to polymerize G-actin [33] and H2A-H2B histone dimer to bundle F-actin filaments [34]. H2A histone is protected from proteolysis by actin and actin inhibits the antimicrobial activity of H2A histone [27]. Importantly, histones were found in the bronchopulmonary secretions of patients with cystic fibrosis [35]. H1 histone, which was shown to be present in cystic fibrosis sputum [36], may induce there the formation of F-actin and DNA bundles, which increases the viscosity of the sputum. The above results show that extracellular histones interact with actin in a pathologically significant way. However, the details of histone-actin interactions, such as kinetics of bundle formation of F-actin, characterization of their interactions in molecular detail, and the effect of several factors impacting the structure and stability of actin-histone bundles remain uncharacterized.

In this study, we used a histone mixture preparation, which represents histones secreted from the cells during apoptosis. We used also H2A histone with known molecular characteristics including sequence (SGRGKQGGKARAKAKSRSSRAGLQFPVGRVHRLLRKGNYSERVGAGAPVYLAAVLEYLTAEILELAGNAARDNKKTRIIPRHLQLAIRNDEELNKLLGRVTIAQGGVLPNIQAVLLPKKTESHHKAKGK), molecular mass (14000 Da) and structure to investigate histone-actin interactions. We showed that both histone preparations polymerize G-actin, bundle F-actin and significantly increase its viscosity at substoichiometric or stoichiometric concentrations. We found that several factors, including ionic strength, cofilin, DNase1 and DNA affect the structure and stability of the histone induced F-actin bundles. The results indicate that histones are tightly bound to F-actin by strong electrostatic and hydrophobic interactions and affect actin structure.

## Materials and methods

### Materials

N-(1-pyrene) maleimide, Alexa488 SE were obtained from Molecular Probes (Eugene, OR). DNase1, ATP, ADP, dithiotreitol (DTT), histone type III (histone mixture) and deoxyribonucleic acid (DNA) from calf thymus were purchased from Sigma Chemical Co. (St Louis, MO). Acetone dry powder was purchased from Pel-freeze Biologicals (Rogers, AR). Human recombinant H2A histone was bought from New England Bio Labs (Ipswich, MS). Viscous Aqua was purchased from Ursa BioScience (Abingdon, MD). Yeast cofilin was a generous gift of Prof. Emil Reisler, (University of California, Los Angeles, CA)

### Preparation of actin

CaATP-G-actin was prepared from acetone dried powder derived from the back and leg muscles of rabbit by the method of Spudich and Watt [37] that yields highly homogeneous actin in purity greater than 90%. CaATP-G-actin was stored in a buffer containing 5 mM TrisHCl, 0.2 mM CaCl_2_, 0.2 mM ATP, 0.5 mM β-mercaptoethanol, pH 8.0 (CaATP-G-buffer). MgF-actin was polymerized from CaATP-G-actin by incubation with 2 mM MgCl_2_ at room temperature for 30 min. MgF-actin was diluted for further treatments in MgF-buffer containing 5 mM MOPS, 2 mM MgCl_2_, 0.2 mM ATP and 0.5 mM DTT, pH 7.4. The concentration of unlabeled rabbit skeletal muscle CaATP-G-actin and Mg-F-actin was determined spectrophotometrically using the extinction coefficients *E*1%290nm = 11.5 cm^-1^. (The optical density of actin was measured in the presence of 0.5 M NaOH, which shifts the maximum of absorbance from 280 nm to 290 nm). Molecular masses of skeletal actin, yeast cofilin and H2A histone were assumed to be 42 kDa, 15.9 kDa and 14.1 kDa, respectively.

### Pyrene labeling

Labeling of Mg-F-actin at Cys-374 with pyrene maleimide was carried out according to Kouyama and Mihashi [38] with some modifications [39]. The concentration of modified actin was determined by Bradford’s’ method [40] using unmodified actin as a standard. The extent of labeling was measured by using pyrene extinction coefficient *ɛ*_344 nm_ = 22000 cm^-1^M^-1^ was, 100%.

### Actin labeling with Alexa488 SE

Labeling was carried out according to Mahaffy et al. [41] and Grintsevich et al [42]. Actin was labeled on lysines with Alexa Fluor 488 succinimidyl ester. Actin filaments were dialyzed against labeling buffer (50 mM PIPES, pH 6.8, 50 mM KCl, 0.2 mM CaCl_2_, 0.2 mM ATP) for 3 h to remove DTT and to adjust the pH. Filaments were incubated in labeling buffer with a 3-7-fold molar excess of dye overnight at 4°C. Labeled F-actin was pelleted in TLA110 rotor, 60,000 rpm, for 30 min then resuspended in CaATP-G-buffer, and dialyzed for 48 h versus the same buffer. Labeled actin monomers were centrifuged in TLA110 rotor, 60,000 rpm, 30 min followed by gel-filtration (Superdex S200 10/300 GL). The label concentration was calculated using *ɛ*_495nm_ = 71,000 M^−1^cm^−1^.

### Fluorescence and light scattering measurements

Actin polymerization was followed as increase in fluorescence of pyrene-labeled G-actin [38], which was added to unlabeled G-actin in 10–15%. The time course of pyrene-labeled actin polymerization was monitored by measuring fluorescence increase (with 365 nm excitation and 386 nm emission wavelengths) in a PTI spectrofluorometer (Photon Technology Industries, South Brunswick, NJ). Actin viscosity measurements were carried out using Viscosity Aqua fluorescent viscosity probe (Ursa BioScience, Abingdon, MD). The fluorescence of the probe was measured in a PTI spectrofluorometer at 400 nm excitation and 440–540 nm emission wavelengths and at 492 nm emission maximum. The fluorescence of the probe is increasing with the increase in viscosity. Structural changes including bundling of MgF-actin was followed by light scattering [2]. The time course of light scattering changes was also measured in a PTI spectrofluorometer, with both excitation and emission wavelengths adjusted to 450 nm. All fluorescence, light scattering and sedimentation experiments were carried out at 22 C°.

### Monitoring actin polymerization by high speed and bundling by low speed sedimentation

For monitoring extent of polymerization samples were centrifuged at 129,151xg for two hours (high speed centrifugation) after addition of histone mixture or H2A histone to actin. For monitoring the extent of F-actin bundling samples were centrifuged at 20,800xg for 8 min (low speed centrifugation). The supernatants were run on SDS-PAGE and analyzed by densitometry with TINA 2.07d software.

### TIRF microscopy

Untethered 15% Alexa488 SE labeled actin filaments were imaged on Pluronic F127-coated surface [43]. Single flow chambers (volume~30 μl) were assembled using two layers of permanent double sided Scotch tape. Slides were equilibrated with 3 chamber volumes (CV) of 1xTIRF imaging buffer (10 mM Hepes, 1 mM MgCl2, 50 mM KCl, 0.2 mM EGTA (pH 7.4) supplemented with 50 mM DTT, 0.2 mM ATP, 0.05 mg/ml casein, 20 mM glucose, 0.25 mg/ml glucose oxidase, 50 μM catalase, 0.5% methyl cellulose). For each sample Ca-ATP-G-actin was incubated for 3 min at room temperature with Mg/EGTA exchange buffer (0.1 mM EGTA, 50 μM MgCl_2_). Actin polymerization was started by addition of polymerizing salts and proteins (or corresponding buffers) to Mg-ATP-G-actin; the resulting mixtures (3 CV) were introduced into the flow chambers. After 15–30 min of on-slide polymerization (at room temperature) images of the random fields were taken. All TIRF data was analyzed using ImageJ (Fiji) software (NIH, Bethesda, MD). Background subtraction was done using rolling ball radius algorithm (50 pxls). The images were converted into black-and-white 8-bit format then the colors of the objects and background were inverted for clarity of presentation.

### Statistical analysis

Student’s t test and one-way ANOVA were used for calculation of p values in evaluation of significance. * = p<0.05, ** = p<0.01, *** = p<0.005. Unless specified, all presented data are mean ± SD of three independent experiments performed in triplicate. All presented SDS gels blots are representative of three independent experiments.

## Results

### Histones polymerize G-actin, bundle F-actin and increase F-actin viscosity

**Fig 1:**
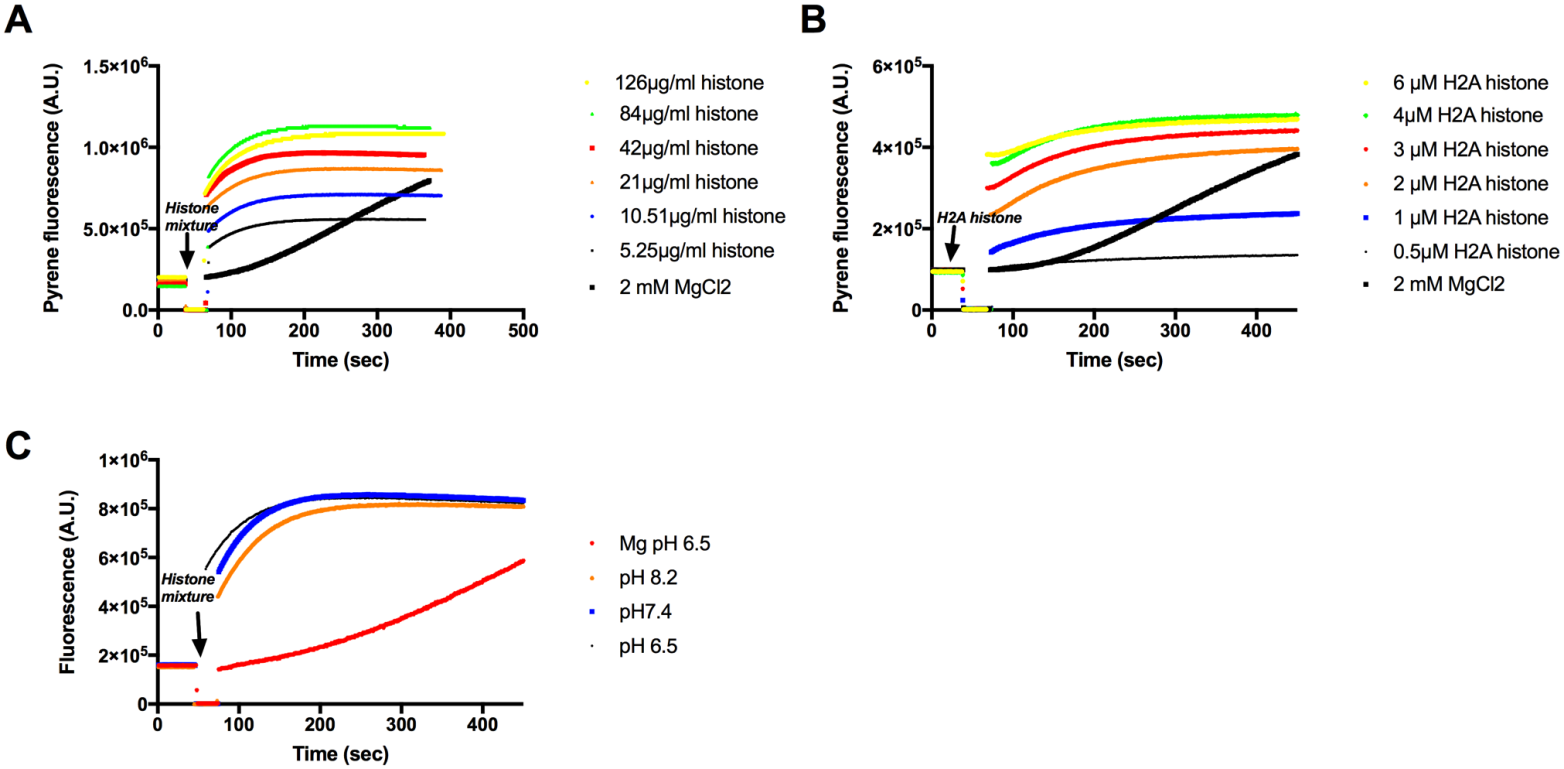
Polymerization of CaATP-G-actin by histone mixture and H2A histone followed by increase in pyrene fluorescence. (A) 5.25–126 μg/ml histone mixture, (B) 0.5–6 μM (7–84 μg/ml) H2A histone, or 2 mM MgCl_2_ were added to pyrene labeled (10% labeling ratio) 4 μM CaATP-G-actin in pH 7.4 CaATP-G-buffer. (C), 21 μg/ml histone mixture was added to pyrene labeled (10% labeling ratio) 4 μM CaATP-G-actin in pH 6.5, 7.4 and 8.2 CaATP-G-buffer or 2 mM MgCl_2_ was added in pH 6.5 CaATP-G-buffer. Fluorescence measurements were carried out as given in MATERIALS and METHODS. Presented data are representative of three independent experiments.

**Fig 2:**
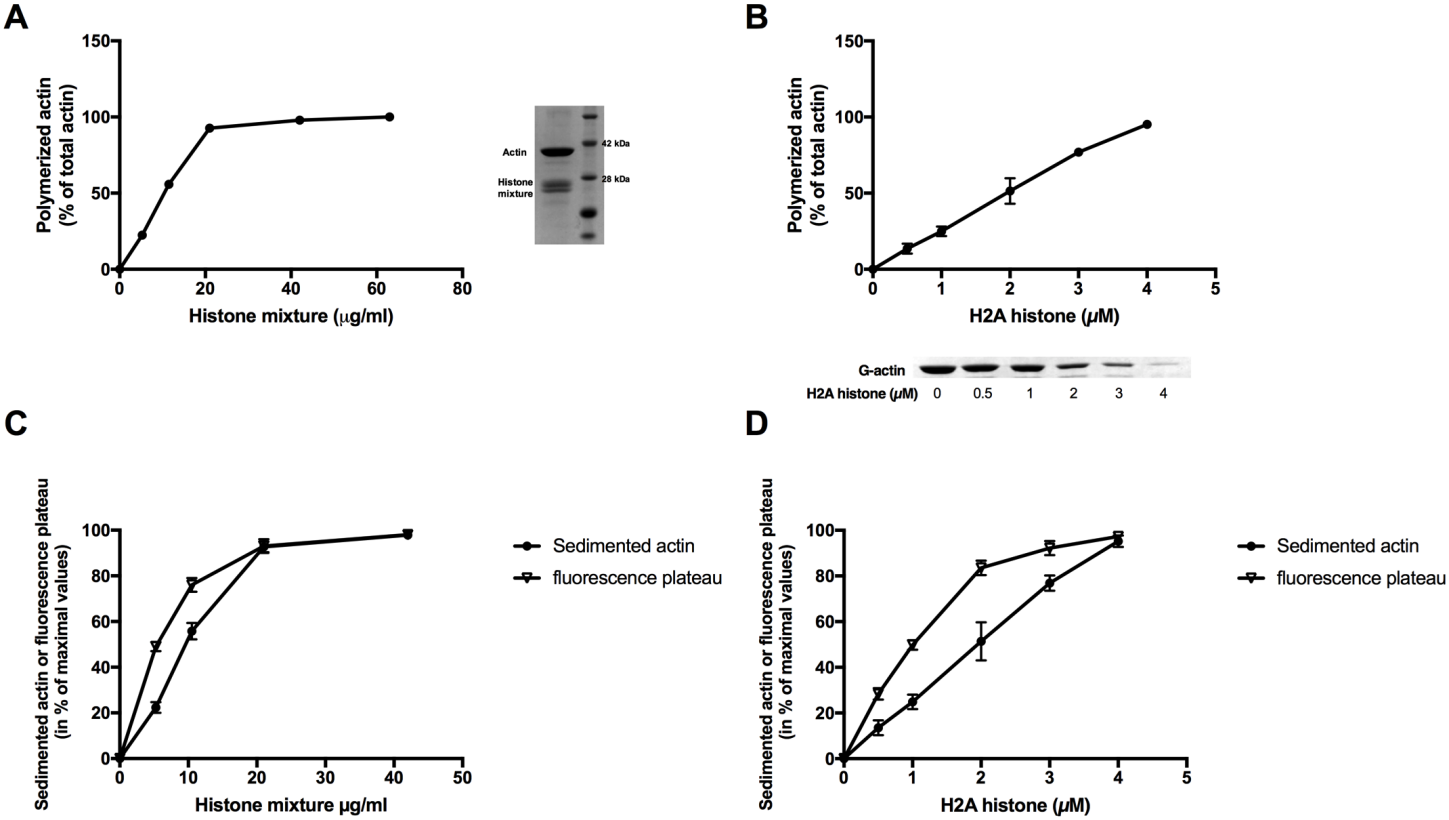
Polymerization of CaATP-G-actin by histone mixture and H2A histone followed by high speed centrifugation was compared with the plateaus of the pyrene fluorescent measurements. (A) 5.25–63 μg/ml histone mixture, or (B) 0.5–4 μM (7–56 μg/ml) H2A histone was added to 4 μM CaATP-G-actin in pH7.4 CaATP-G-buffer. Samples were centrifuged at 129,151xg for 2h, supernatants run on SDS-PAGE and evaluated as described in MATERIALS and METHODS. Fig 2A, inset: SDS-PAGE, left, actin and histone mixture before centrifugation; right, molecular weight marker. Fig 2B, inset: actin lanes from the SDS-PAGE of supernatants after high speed centrifugation. All SDS-PAGE gels are representatives of three independent experiments. Actin sedimentation values were compared with plateaus of pyrene fluorescence upon addition of histone mixture (C) or H2A histone (D). Pyrene fluorescence values were taken from Fig 1. Sedimentation data were taken from experiments presented in Fig 2A and B. The presented data are mean and standard deviation of three independent experiments.

Monovalent, divalent and polyvalent cations are well known to polymerize G-actin to actin filaments. The polymerizing activity of several positively charged polycationic antimicrobial polypeptides, including lysozyme and LL-37 were studied [10], [11]. We found by measuring pyrene fluorescence increase that low concentrations of histone mixture and H2A histone rapidly polymerize 4 μM G-actin to F-actin in a concentration dependent manner (Fig 1A and 1B) even in the absence of added salts. The rate limiting nucleation step of histone induced polymerization of actin is faster than that of the conventional Mg induced polymerization (Fig 1A and 1B). Polymerization of actin by histone mixture is pH independent in the 6.5–8.2 pH range (Fig 1C). The histone concentration dependence of polymerization was studied by high speed sedimentation. We found that 21 μg/ml histone mixture or 4μM (56 μg/ml) H2A histone were necessary for maximal polymerization of 4 μM CaATP-G-actin (Fig 2). We cannot determine the exact molecular weight of histone mixture since it composed of several histones with different molecular weights. However, it is possible to use an average molecular weight of 21K according to molecular weight marker (Fig 2A inner panel). Accordingly the 21 μg/ml histone mixture is 1 μM, i.e. it is substoichiometric relative to actin. From H2A histone stoichiometric concentration is needed to fully polymerize actin. The low concentrations of histones, relative to G-actin, needed for polymerization may indicate that histones stabilize actin dimer, which is the most labile intermediate in the process of actin polymerization. The amount of actin sedimented at various histone concentrations was compared to the plateau of pyrene fluorescence obtained at the same histone concentration (Fig 2C and 2D). The plateau values increased more than the amount of actin sedimented with the concentration of histone mixture or H2A histone added. This indicates that the increase in pyrene fluorescence is not caused solely by the polymerization of actin into filaments but also by internal structural changes taking place in actin filaments during the polymerization process.

**Fig 3:**
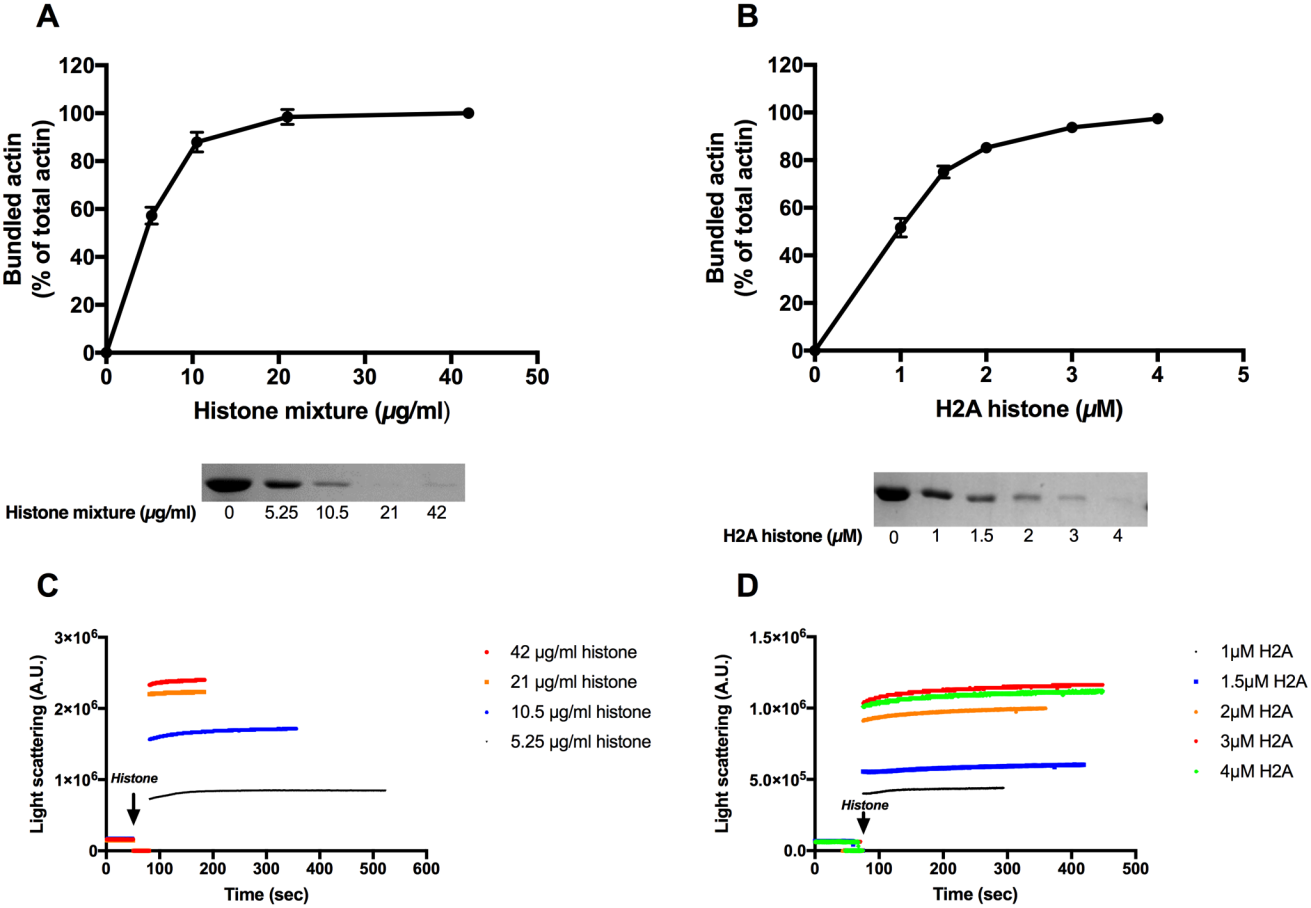
Histone mixture and H2A histone induced bundle formation of Mg-F-actin followed by low speed centrifugation and by light scattering. 5.25–84 μg/ml histone mixture (A), or 1–4 μM (14–56 μg/ml) H2A histone (B), were added to 4 μM MgF-actin in pH7.4 F-buffer and centrifuged at low speed. Samples were centrifuged at 20,800xg for 8 min, supernatants run on SDS-PAGE and evaluated as described in MATERIALS and METHODS. The presented data are mean and standard deviation of three independent experiments. Insets: actin lanes, representatives of three independent experiments, from SDS-PAGE of low speed centrifugation supernatants. (C) 5.25–42 μg/ml histone mixture or (D) 1–4 μM (14–56 μg/ml) H2A histone were added to 4 μM MgF-actin in pH7.4 F-buffer and the light scattering change was followed as described in MATERIALS and METHODS. Presented data are representative of three independent experiments. All measurements were done at pH7.4 in F-buffer.

**Fig 4:**
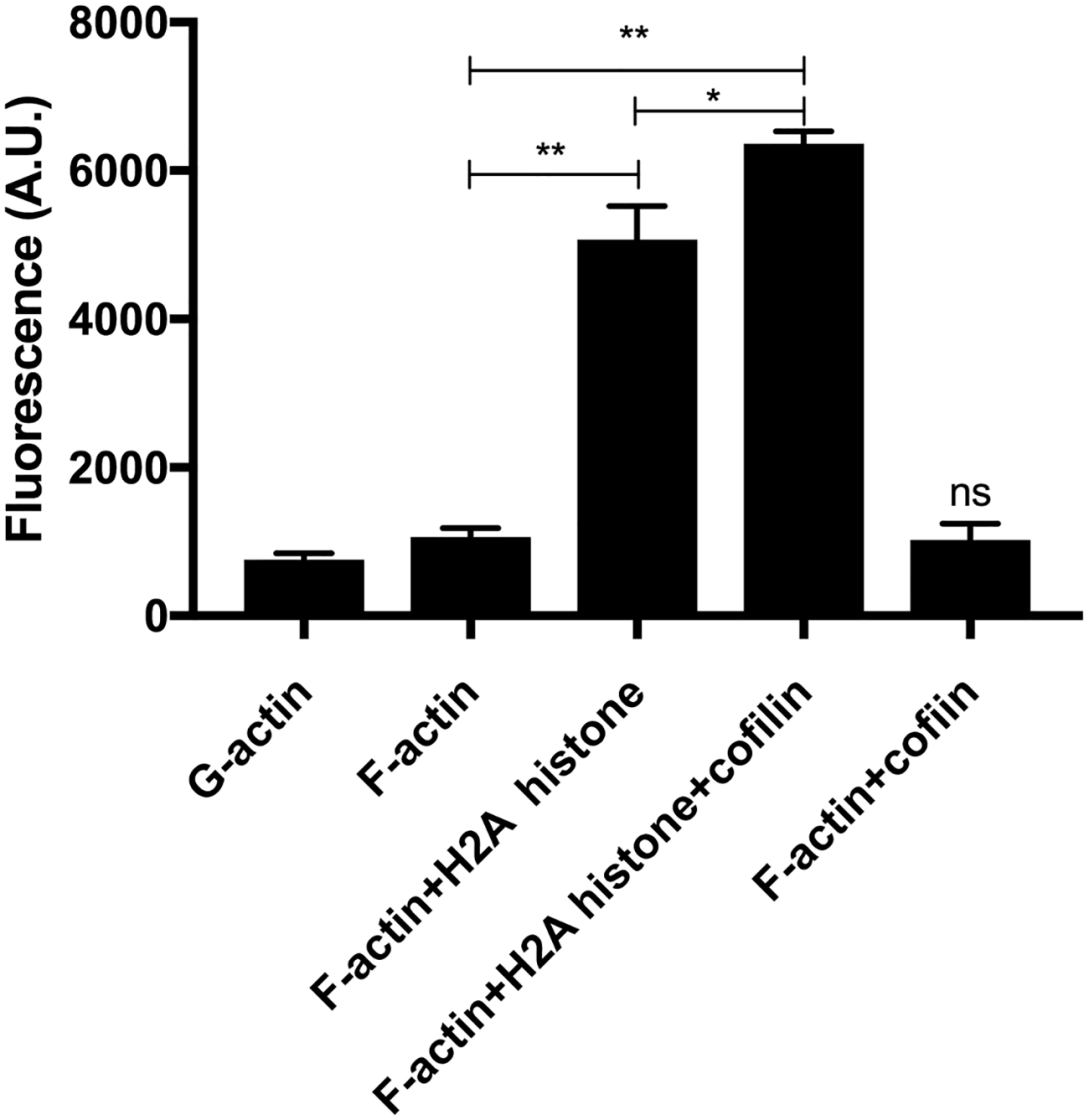
Effect of 3 μM (42 μg/ml) H2A histone and 6 μM cofilin on the viscosity of 4 μM MgF-actin measured by Viscous Aqua fluorescence viscosity probe. Viscous Aqua in original Ursa BioScience vial was dissolved in 50 μl methanol then diluted 50 times in actin buffer and added to actin containing solutions in 1 to 50 ratio in pH 7.4 buffer. The fluorescence of the mixtures was measured as described in MATERIALS and METHODS. The fluorescence values (in artificial units, A.U.) of the samples at 492 nm emission maximum minus the fluorescence of the buffer are given in the figure. The data obtained were compared by statistical analysis and the significance of the differences was indicated. * = p<0.05, ** = p<0.01 *** = p<0.005. The fluorescence emission increases with the increasing viscosity of the samples. The presented data are mean and standard deviation of at least three independent experiments.

F-actin was shown to form bundles upon addition of polyvalent cations [2] because the positive charged cations eliminate the repulsion between the negatively charged actin filaments [3]. We monitored the histones induced F-actin bundling by low speed centrifugation since bundled actin filaments are sedimented at low speed while unbundled actin filaments only at high speed centrifugation. We found that 21 μg/ml (1 μM) histone mixture (Fig 3A) and 3 μM (42 μg/ml) H2A histone (Fig 3B) fully bundle (98% and 94% bundling) of 4 μM F-actin. The kinetics of histone induced F-actin bundling was followed light scattering. Light scattering results should be interpreted with caution because they might be caused not only by bundling but also by other structural effects as change in the shape of the scattering object. In this case it is obvious that the scattering increase is caused by filaments bundling as it was also indicated by low speed centrifugation (Fig 3A and 3B) and TIRF microscopy. We found that 21 μg/ml (1 μM) histone mixture (Fig 3C) or 3 μM (42 μg/ml) H2A histone (Fig 3D) induce fast maximal light scattering increase in 4 μM F-actin. Same histone concentrations were shown to cause near complete bundling of F-actin in low speed centrifugation experiments (Fig 3A and 3B). Both histone mixture and H2A histone concentrations needed for bundling are substoichiometric relative to F-actin and indicate tight binding of histones to actin filaments. Addition of 3μM H2A (42 μg/ml) histone to 4 μM F-actin was found to greatly increase (by 296%) the viscosity of the F-actin containing solution (Fig 4), which may have pathophysiological significance in the cystic fibrosis disease.

### Effect of NaCl, DNase1, DNA and cofilin on histone induced actin bundles

**Fig 5:**
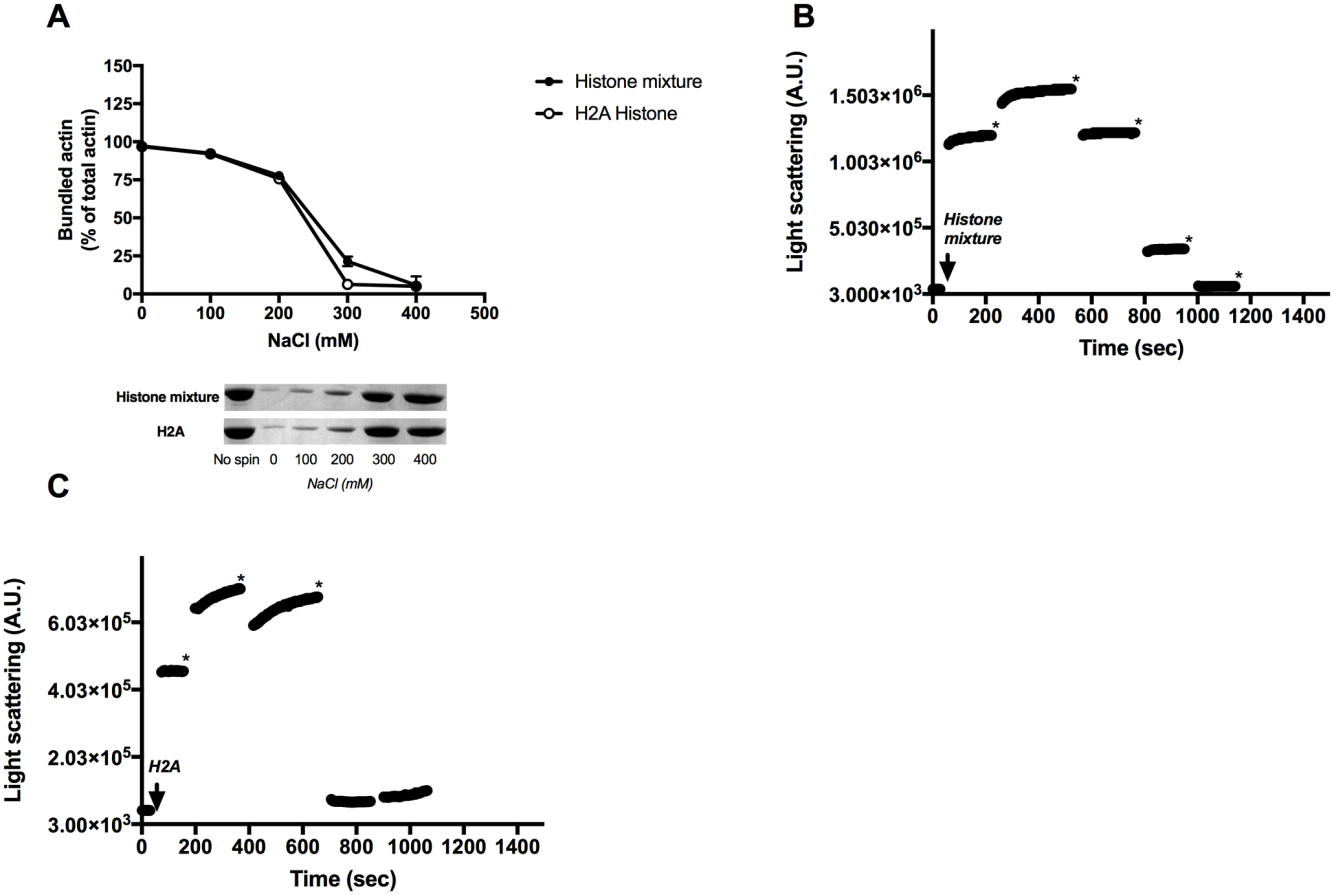
Effect of 0–400 mM NaCl on the sedimentation of 63 μg/ml histone mixture or 4 μM (56 μg/ml) H2A histone bundled 4 μM MgF-actin as measured by low speed centrifugation and light scattering. Sedimentation: (A), Bundling by histone mixture and H2A histone. Samples were centrifuged at 20800xg for 8 min, supernatants run on SDS-PAGE and evaluated as described in MATERIALS and METHODS. The presented data are mean and standard deviation of three independent experiments. Insets: actin lanes, representatives of three independent experiments, from the SDS-PAGE of supernatants after low speed centrifugation. Light scattering: (B), 4x100 mM NaCl was added to histone mixture bundled 4 μM MgF-actin, (C), 3x100 mM NaCl was added to H2A histone bundled 4 μM MgF-actin and the light scattering was measured. Asterisks* represent 100 mM NaCl addition. Light scattering change was followed as described in MATERIALS and METHODS. Presented data are representative of three independent experiments.

Polycation induced F-actin bundles are sensitive to ionic strength and unbundle (dissociate) with increase in the concentration of monovalent cations [2] because they mask the electrostatic interactions between the polycations and the negatively charged actin filaments [3], [44]. The salt sensitivity of the polycation induced F-actin bundles provides insight into the relative strength of electrostatic and hydrophobic interactions in the binding of polycations to F-actin. Low ionic strength sensitivity indicates a significant role of the hydrophobic interactions in the binding of the polycations to the filaments. We found in low speed centrifugation experiments that H2A histone and histone mixture induced F-actin bundles are completely dissociated only in the presence of 300 and 400mM NaCl, respectively (Fig 5A), indicating relatively strong hydrophobic interactions between F-actin and histones. Similarly 300–400 mM NaCl was needed to get maximal decrease in the light scattering of histone mixture (Fig 5B) and H2A histone (Fig 5C) induced actin filament bundles. Interestingly addition of 100 mM NaCl does not decrease but increase the light scattering of the system (Fig 5). This cannot indicate increased bundling since in the absence of added NaCl the actin is almost completely bundled but salt induced structural changes in F-actin. It is worth to mention that F-actin bundles induced by other polycations, such as polylysine, spermine, and lysozyme disassemble already at 100 mM [2] and LL-37 at 200 mM NaCl [10] concentrations. The comparison of these results with those obtained with histone induced bundles supports the existence of relatively strong hydrophobic interactions between histones and F-actin. However, the high net positive charge content of histones, H2A histone has 14 net positive charges, certainly also contributes to the strength of histone-F-actin interactions.

**Fig 6:**
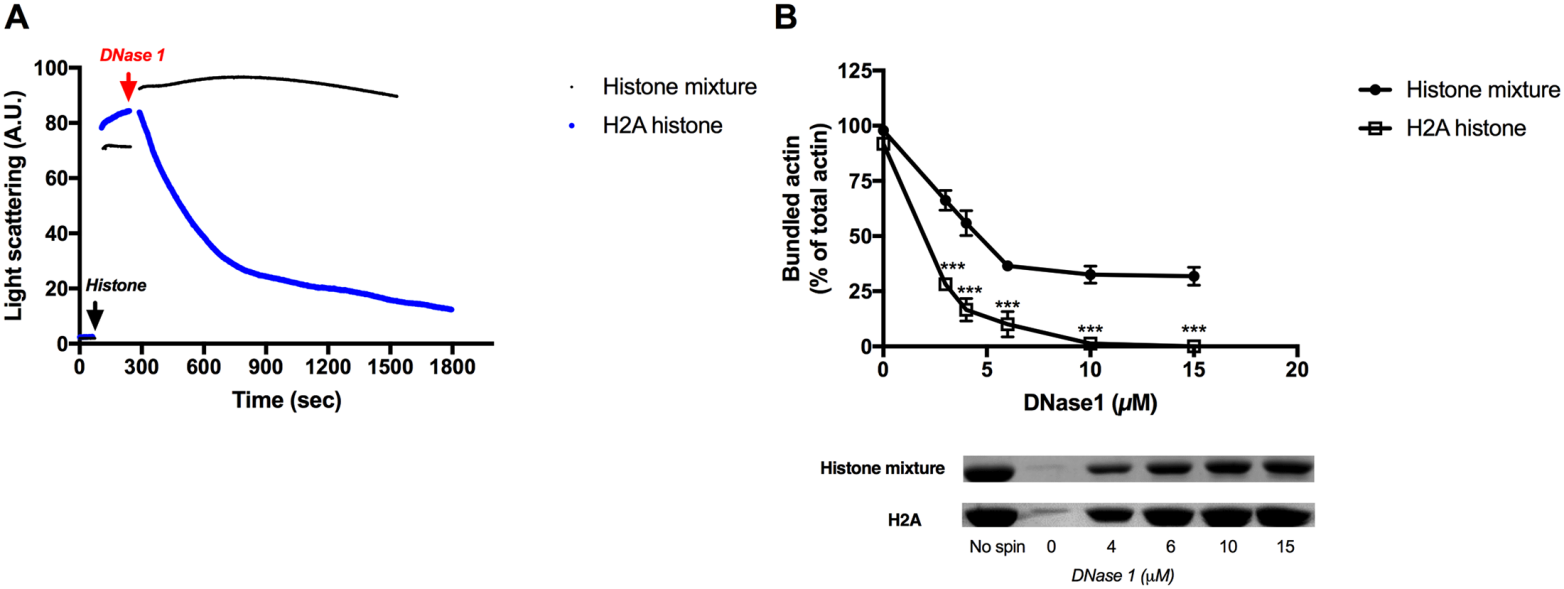
Effect of DNase1 on the light scattering and sedimentation of 4 μM MgF-actin bundled by histone mixture or H2A histone. (A) Effect of 9 μM DNase1 on the light scattering of 4 μM MgF-actin bundled by 63 μg/ml histone mixture or 3 μM (42 μg/ml) H2A histone. Light scattering change was followed as described in MATERIALS and METHODS. Presented data are representative of three independent experiments. (B). Effect of 2–15 μM DNase1 on the sedimentation of 4 μM MgF-actin bundled by 63 μg/ml histone mixture or 3 μM (42 μg/ml) H2A histone. The difference between the amount of actin sedimented following DNase1 treatment of histone mixture and H2A histone bundled actin is highly significant. Samples were centrifuged at 20800xg for 8 min, supernatants run on SDS-PAGE and evaluated as described in MATERIALS and METHODS. The presented data are mean and standard deviation of three independent experiments. Insets: actin lanes, representatives of three independent experiments, from SDS-PAGE of low speed centrifugation supernatants.

DNase1 is a G-actin binding protein. It binds very tightly to the D-loop of G-actin and forms co-crystals with actin monomers [45] and depolymerizes F-actin by binding to the protomers at the filaments ends. We found that it efficiently dissociates LL-37 induced F-actin bundles [10]. Here we studied the effect of DNase1 on F-actin bundled by histones. In light scattering experiments 9 μM DNase1 very slowly dissociates histone mixture bundled and much faster H2A histone bundled (Fig 6A) F-actin. The reason in the difference in the dissociation kinetics could be that the binding of DNase1 to the ends of H2A bundled filaments is faster than to the histone mixture bundled filaments. In sedimentation experiments increasing concentration of DNase1 was added to 4 μM F-actin bundled by 63 μg/ml histone mixture or 3 μM (42 μg/ml) H2A histone and after 30 min incubation it was subjected to low speed centrifugation (Fig 6B). The amount of bundled actin decreased with the increasing DNase1 concentration. H2A histone bundled filaments fully depolymerized by 10 μM DNase1. However, the depolymerization of histone mixture bundled F-actin was not complete even after the addition of 15 μM DNase1 (about 35% of F-actin still sedimented under this condition) (Fig 6B). The difference in the amount of actin sedimented of the histone mixture bundled and of the H2A histone bundled F-actin following DNase treatment is statistically highly significant. The difference between the effect of DNase1 on the light scattering of H2A histone and histone mixture bundled actin is much larger than the difference between the effect of DNase1 on the sedimentation of F-actin bundled by the two histones. The reason of the differences is that the light scattering depends also on structural changes and not only on the extent of bundling. It seems that the structure of the histone mixture induced F-actin bundle is different from that of the H2A histone induced one; therefore, DNase1 causes less decrease in the light scattering of F-actin bundled by histone mixture than bundled by H2A histone. The results of the light scattering (Fig 6A) and the low speed sedimentation (Fig 6B) experiments indicate tight histone mixture binding to F-actin. Both the light scattering and the sedimentation of the H2A histone bundled F-actin is more sensitive to DNase1 than F-actin bundled by histone mixture. This indicates that the binding of H2A histone to F-actin is less tight than that of the histone mixture.

**Fig 7:**
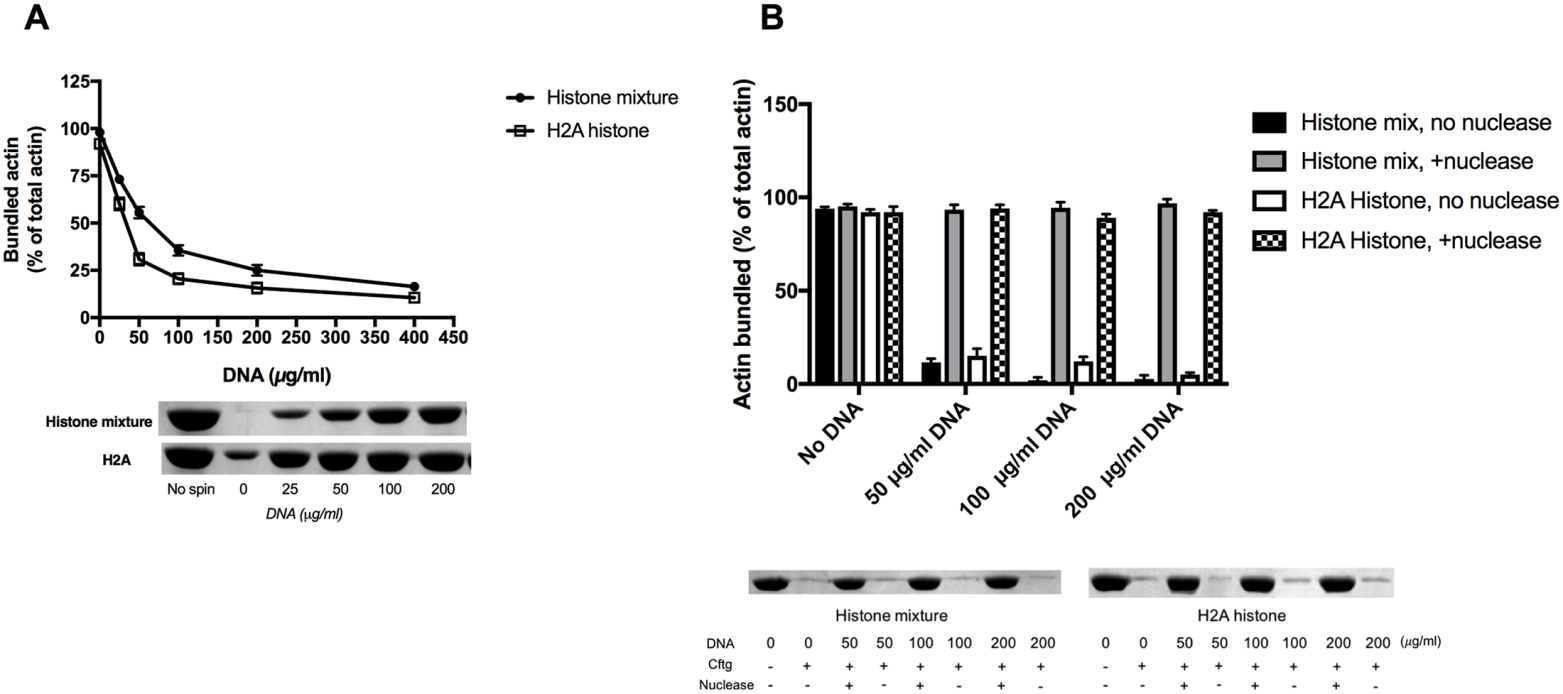
Effect of DNA on the bundling of F-actin by histone. (A), Effect of 25–400 μg/ml DNA on the sedimentation of 4 μM MgF-actin bundled by 42 μg/ml histone mixture or 3 μM (42 μg/ml) H2A histone. (B), Effect of 0–200 μg/ml DNA digested by Staphylococcus aureus micrococcal DNase on the sedimentation of 4 μM MgF-actin bundled by 42 μg/ml histone mixture or by 3 μM (42 μg/ml) H2A histone. 4 μg/ml DNA was digested by 20 μg/ml micrococcal DNase at 37°C for 30 min. Samples were centrifuged at 20800xg for 8 min, supernatants run on SDS-PAGE and evaluated as described in MATERIALS and METHODS. The presented data are mean and standard deviation of three independent experiments. Insets: actin lanes, representatives of three independent experiments, from the SDS-PAGE of low speed centrifugation supernatants.

Histones form together with DNA well defined tight structures in the cell nucleus and are secreted from the cell following apoptosis. Since we have shown that histones are strongly bound also to F-actin, it was of interest to study the competition between DNA and actin for histones. Because of this competition DNA “removes” histone from F-actin-histone bundles, which results the dissociation of the bundles. The effect of DNA on histones induced bundling was evaluated by low speed centrifugation experiment (Fig 7A). Addition of 200 μg/ml DNA to 42 μg/ml histone mixture and 3 μM (42 μg/ml) H2A histone bundled 4 μM F-actin decreased the amount of F-actin sedimented to 18% and 15% of that obtained in the absence of DNA, respectively. The amount of sedimented actin decreased more upon addition of DNA to H2A histone bundled F-actin than to histone mixture bundled F-actin. This again shows that the binding of H2A histone to F-actin is less tight than that of the histone mixture. We also studied the effect of digestion of DNA on its ability to dissociate histone induced F-actin bundles by low speed centrifugation. In these experiments DNA, cleaved to oligomers by *Staphylococcus aureus* nuclease, was added to histone mixture or histone 2A bundled F-actin (Fig 7B). The effect of DNA digestion had a dramatic effect: nuclease cleaved DNA up to 200 μg/ml concentration did not unbundle neither histone mixture nor H2A histone bundled F-actin. This indicates that not only the negative charge but also the macromolecular structure of DNA is a requirement for dissociation F-actin bundles.

**Fig 8:**
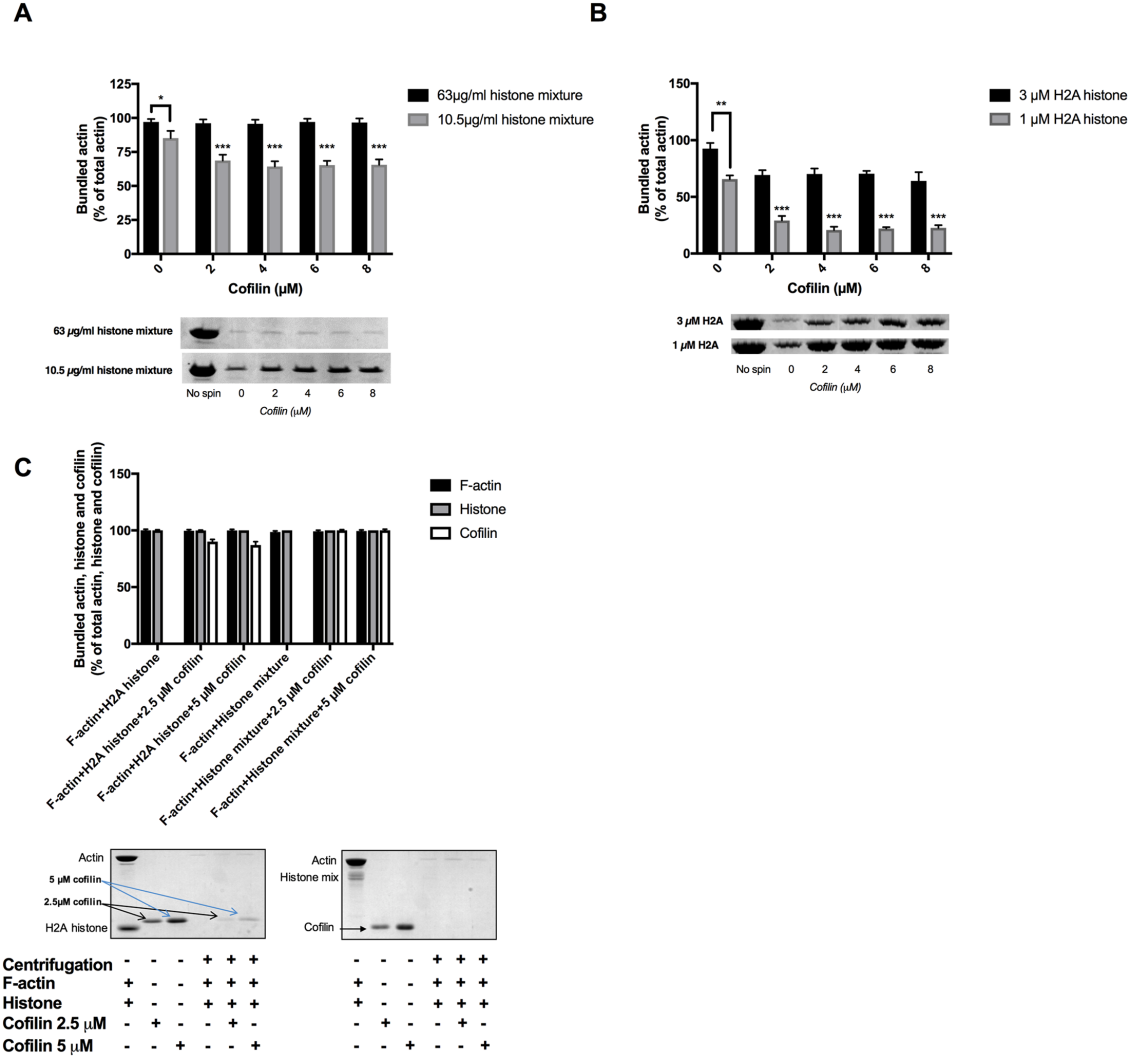
Effect of cofilin on the sedimentation of 4 μM MgF-actin bundled by histone mixture or H2A histone. (A), 2–8 μM cofilin added to 4 μM F-actin bundled by 10.5 and 63 μg/ml histone mixture or (B) by 1 μM (14 μg/ml) and 3 μM (42 μg/ml) H2A histone. (C), 42 μg/ml histone mixture or 4 μM (56 μg/ml) H2A histone and 2.5 or 5 μM cofilin were added simultaneously to 4 μM F-actin. Samples were centrifuged at 20800xg for 8 min, supernatants run on SDS-PAGE and evaluated as described in MATERIALS and METHODS. The presented data are mean and standard deviation of three independent experiments. Insets: lanes of SDS-PAGE gels, representatives of three independent experiments, obtained from SDS-PAGE of low speed centrifugation supernatants.

**Fig 9:**
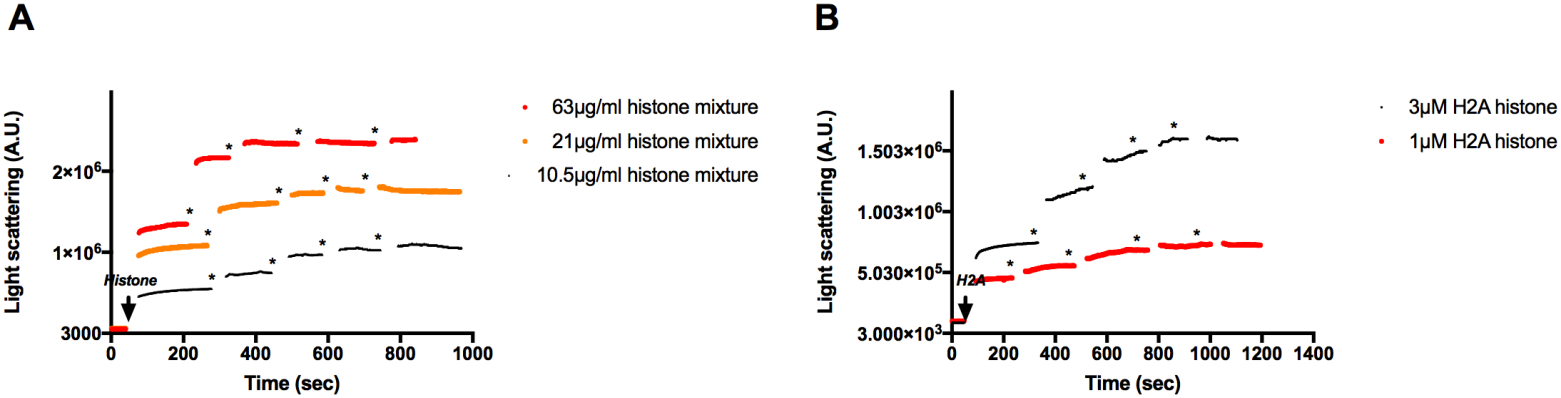
Effect of cofilin on the light scattering of histone mixture or H2A histone bundled MgF-actin. 4x 2 μM cofilin was added to 4 μM MgF-actin bundled by 63 μg/ml histone mixture (A), or by 4 μM (56 μg/ml) H2A histone (B). Stars indicate addition of 2 μM cofilin. Light scattering change was followed as described in MATERIALS and METHODS. Presented data are representative of three independent experiments.

**Fig 10:**
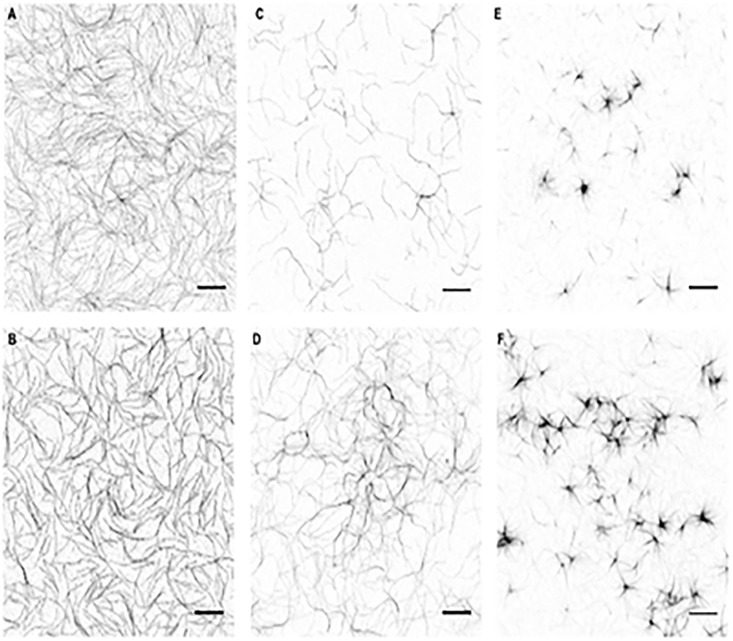
TIRF microscopy revealed a change in morphology of histone-induced F-actin bundles in the presence of cofilin. In all experiments actin was polymerized by 1 mM MgCl_2_ containing in TIRF imaging buffer. (A), Actin (1μM) polymerized in TIRF imaging buffer for 15 minutes forms unbundled long filaments. (B), Actin (1μM) copolymerized with 1 μM cofilin in TIRF imaging buffer for 15 minutes. More short filaments were detected in the sample due to the cofilin-induced severing. (C-D), Actin (1μM) copolymerized with 1 μM (14 μg/ml) H2A histone in TIRF imaging buffer for 15 (C) and 30 minutes (D), respectively. Thin, cable-like F-actin bundles were detected. (E-F), Actin (1μM) copolymerized with 1 μM (14 μg/ml) H2A histone and 1 μM cofilin in TIRF imaging buffer for 15 (E) and 30 minutes (F). Star-like F-actin structures were detected under these conditions. Scale bars: 10 μm. Presented data are representative of three independently taken microscope images. TIRF imaging buffer: 10 mM Hepes, 1 mM MgCl2, 50 mM KCl, 0.2 mM EGTA (pH 7.4) supplemented with 50 mM DTT, 0.2 mM ATP, 0.05 mg/ml casein, 20 mM glucose, 0.25 mg/ml glucose oxidase, 50 μM catalase, 0.5% methyl cellulose. TIRF microscopy and Alexa 484 SE labeling were carried out as described in MATERIALS and METHODS.

Cofilin is an actin filament severing protein [46]. It cuts actin filaments into shorter segments but does not depolymerize directly F-actin to G-actin monomers. Due to its severing effect cofilin dissociates bundles because of the decreased affinity of shorter actin filaments to react with each other, as we found studying the effect of cofilin on polylysine, spermine, lysozyme [2] and LL-37 [10] induced actin bundles. Here we examined the influence of cofilin addition on histones induced actin bundles by low speed centrifugation (Fig 8A & 8B), light scattering (Fig 9A & 9B) and TIRF microscopy (Fig 10) experiments. In low speed centrifugation experiments the addition of cofilin either did not affect the amount of F-actin sedimented, when it was bundled by 63 μg/ml, or slightly decreased, when it was bundled by 10.5 μg/ml histone mixture (Fig 8A). Cofilin decreased the sedimentation of F-actin bundled by both 1 μM and 3 μM H2A histone (Fig 8B). The decrease in the amount of F-actin sedimented was less when it was bundled with high than with low H2A histone concentration, which causes only partial bundling, probably because the bundled actin has higher resistance to the severing action of cofilin than that of unbundled actin filaments [47]. We added 2.5 or 5 μM cofilin and 42 μg/ml histone mixture or 4 μM H2A histone simultaneously to 4 μM F-actin and found by low speed centrifugation that both histones and cofilin are bundled together with actin filaments (Fig 8C) indicating the simultaneous binding of histones and cofilin to F-actin. We studied also the effect of cofilin on the viscosity of H2A bundled F-actin and found that cofilin further significantly increases by 24 percent the already high the viscosity of the system (Fig 4). In light scattering measurements cofilin does not decrease but increases the light scattering of F-actin bundled both by histone mixture (Fig 9A) and H2A histone (Fig 9B). The 2 μM cofilin induced scattering increase was observed also when 4 μM F-actin was bundled only by 10.5 μg/ ml histone mixture (Fig 9A) or 1 μM (14 μg/ml) H2A histone (Fig 9B), which only partially bundles actin filaments. Light scattering increase does not necessary indicate increased bundling of actin filaments but may signal other structural changes in the system. To investigate these possible structural changes of the histone bundled actin filaments we studied the system also by TIRF microscopy (Fig 10). In these experiment 1 μM MgATP-G-actin in slide polymerized by 1 mM MgCl_2_ for 15 minutes (Fig 10A), copolymerized by 1 mM MgCl_2_ and 1 μM cofilin for 15 minutes (Fig 10B), by 1 mM MgCl_2_ and 1 μM (14 μg/ml) H2A histone for 15 minutes (Fig 10C) and 30 minutes (Fig 10D), by 1 mM MgCl_2_, 1 μM (14 μg/ml) H2A histone and 1 μM cofilin for 15 minutes (Fig 10E) and 30 minutes (Fig 10F), respectively. The usual meshwork of actin filaments was obtained when G-actin was polymerized for 15 minutes by Mg only (Fig 10A). Upon copolymerization with Mg and cofilin for 15 minutes (Fig 10B) many short actin filaments appear in addition to normal length filaments due to the severing activity of cofilin. On copolymerization with Mg and H2A histone for 15 minutes there are relatively few free and bundled actin filaments (Fig 10C) and the extent of bundling is higher after 30 minutes incubation (Fig 10D). When G-actin was copolymerized with Mg, H2A histone in the presence of cofilin for 15 minutes (Fig 10E) many free short filaments and a few star like dense superstructures appeared on the screen. The number of superstructures was increased after 30 minutes incubation (Fig 10F). The superstructures seem to be formed by great number of short filaments bundled together. The formation of these superstructures may cause the light scattering and viscosity increase observed upon addition of cofilin to histone bundled F-actin (Figs 9 and 4). These results may indicate that cofilin affects either the structure of the individual actin filaments or that of the histone induced actin bundles in such a way that the addition of cofilin leads to the formation of star like superstructures and increases the light scattering and the viscosity of the system.

## Discussion

Histone mixture and H2A histone rapidly bundle F-actin in substoichiometric and stoichiometric concentrations relative to actin. These results point to a tight binding between actin and histones, which may be due to strong electrostatic interactions (H2A histone has 14 net positive charges) and/or to additional hydrophobic interactions. Lower concentration of histones are needed for actin bundling than of other polycations as, LL-37, lysozyme [10], polylysine and spermine [2]. Polycation induced F-actin bundles dissociate upon increase of ionic strength because the added monovalent cations mask electrostatic interactions between the polycations and the negatively charged actin filaments [3], [44]. Lysozyme, polylysine and spermine induced actin bundles dissolve already upon addition of 100 mM NaCl [2], LL-37 induced bundles dissociate in the presence of 200 mM NaCl [10], while bundles induced by histones fully disassemble only in the presence of 300–400 mM NaCl. These results show that histone-F-actin-bundles are more stable than those formed by LL-37, lysozyme, polylysine or spermine. Hydrophobic interactions may contribute to the binding of histones to F-actin as indicated by the relatively low ionic strength sensitivity of the histone induced actin bundles.

The mechanism of action of agents affecting the stability and structure of F-actin–histone bundles was studied. These agents affect the bundles by different mechanisms. NaCl increases ionic strength and dissolves the bundles by masking electrostatic interactions between histones and actin filaments [3], [44]. DNA dissociates bundles by removing histones from F-actin via competition with their binding to F-actin. DNase 1 depolymerizes F-actin filaments to G-actin by dissociating actin monomers from the end of the filaments [48]. Cofilin severs the actin filaments to shorter pieces and decrease their stability by increasing the critical concentration of actin [49]. We found that cofilin decreases the amount of actin sedimented in low speed centrifugation and increases the light scattering of the bundles in a concentration dependent manner. The light scattering increase may indicate structural changes in the histone bundled actin filaments. The possible structural changes in the filament bundles were studied by TIRF microscopy, which showed the formation of star like superstructures upon addition of cofilin. These results could be explained by assuming that while cofilin reduces the amount of actin in the bundles through its severing and depolymerizing action but on the other hand affects the structure of the remaining bundles making them denser with the formation of star like superstructures and, therefore, increases the light scattering of the system. The superstructures consist of large number of short filaments, which seem to attach to each other in a common centrum. Two actin filaments can attach to each other in a single point through their lower dimer (LD) subunits and form an x-like structure as described by Silvan et al. [50]. However, in our case not two but many short filaments join to each other seemingly in a single point. Formation of cofilin-actin rods was described in the distal neurites of neurons [16] and in muscle cells with nemaline myopathy [51]. Cofilin-actin rods destroy neurites in the brain neurons and this effect is assumed to be closely associated with the pathology of the Alzheimer disease [17], [52]. Cofilin oxidation and ATP depletion were found to be necessary conditions for the formation of cofilin-actin rods both in vivo and in vitro [16]. This cannot be the mechanism of the star-like superstructure formation in the present work, since the presence of DTT and ATP in the buffer prevents cofilin oxidation and ATP depletion, respectively. A probable mechanism of the formation of star-like structures under reducing conditions could be that histone bundles the cofilin severed short actin filaments into these structures. The more detailed description of these superstructures and the mechanism of their formation should be the subject of further studies. No superstructure formation was detected with LL-37 or lysozyme induced F-actin bundles, where addition of cofilin decreased both the amount of sedimented actin and light scattering [10] indicating that the structure of these bundles is different from those obtained with histones and cofilin.

Two histone preparations were used in the present experiments. Histone mixture, which represents histone released from dead cells following apoptosis and NET formation [23], and recombinant H2A histone, represents the H2A histone family. The interaction of the two histone preparations with actin was compared. The affinity of histone mixture to F-actin appears to be higher than that of H2A histone because less histone mixture is needed to bundle actin than H2A histone and the dissociation of histone mixture bundled actin filaments by DNase1 is much slower and less complete than that of H2A histone bundled F-actin. Cofilin also has less dissociating effect on histone mixture than on H2A histone induced F-actin bundles. These results indicate also apparent structural differences between histone mixture and H2A histone induced actin bundles.

The high stability and viscosity of the histones induced actin bundles may have pathophysiological significance in cystic fibrosis, which is the most common fatal inherited disease in the western world [15]. Actin bundles had been indicated to contribute the formation of the high viscosity sputum in cystic fibrosis [53]. Large quantities of F-actin released from lysed inflammatory cells are found in the surface airway liquid [54]. Histones, which promote the formation of stable F-actin bundles, were also shown to be present in the bronchopulmonary secretions of patients with cystic fibrosis [35] and [36]. Histones promote the formation of F-actin bundles by neutralizing the repulsive negative charges of the F-actin polyanion [44].The stability of these bundles is higher than those formed by LL-37 or by lysozyme, which are more sensitive to the increase in ionic strength and readily dissolve by DNase1 [10]. These results indicate that extracellular histones might have more important role in formation of sputum in cystic fibrosis than that of LL-37 or lysozyme. The stable histone-actin bundles increase the viscosity of sputum and may aggravate the symptoms of cystic fibrosis patients.

The actin histone interaction might have significance also in cell physiology. The binding of histones to nuclear actin could affect gene activation. It was reported that monomer nuclear actin inhibits while polymer nuclear actin increases the activity of class 1 histone deacetylase [20]. Histone deacetylase removes negatively charged acetyl moieties from histone and, therefore, increases the overall positive charge of the molecule. This allows for tighter winding of DNA and prevents transcription factor binding and gene activation [20]. The direct interaction of actin with histone, described in this paper, decreases the overall positive charge of histones and leads to competition between DNA and actin for histones (Fig 7). As a consequence of this competition less histone attaches to DNA, which can cause less tight winding of DNA and might affect gene expression.
